# Interbrain Synchrony of Team Collaborative Decision-Making: An fNIRS Hyperscanning Study

**DOI:** 10.3389/fnhum.2021.702959

**Published:** 2021-07-15

**Authors:** Mingming Zhang, Huibin Jia, Guanghai Wang

**Affiliations:** ^1^Department of Psychology, College of Education, Shanghai Normal University, Shanghai, China; ^2^Department of Psychology, Henan University, Kaifeng, China; ^3^Key Laboratory of Child Development and Learning Science, Ministry of Education, Southeast University, Nanjing, China; ^4^Department of Developmental and Behavioral Pediatrics, Shanghai Children’s Medical Center, Pediatric Translational Medicine Institute, Shanghai Jiao Tong University School of Medicine, Shanghai, China

**Keywords:** team collaborative decision-making, fNIRS, hyperscanning, sex effects, interbrain synchrony

## Abstract

In many situations, decision-making behaviors are mostly composed of team patterns (i.e., more than two persons). However, brain-based models that inform how team interactions contribute and impact team collaborative decision-making (TCDM) behavior, is lacking. To examine the neural substrates activated during TCDM in realistic, interpersonal interaction contexts, dyads were asked to model TCDM toward their opponent, in a multi-person prisoner’s dilemma game, while neural activity was measured using functional near infrared spectroscopy. These experiments resulted in two main findings. First, there are different neural substrates between TCDM and ISDM, which were modulated by social environmental cues. i.e., the low incentive reward yielded higher activation within the left inferior frontal gyrus (IFG), in individual separately decision-making (ISDM) stage while the dorsolateral prefrontal cortex (DLPFC) and the middle frontopolar area was activated in TCDM stage. The high incentive reward evoked a higher interbrain synchrony (IBS) value in the right IFG in TCDM stage. Second, males showed higher activation in the DLPFC and the middle frontopolar area during ISDM, while females evoked higher IBS in the right IFG during TCDM. These sex effects suggest that in individual social dilemma situations, males and females may separately depend on non-social and social cognitive ability to make decisions, while in the social interaction situations of TCDM, females may depend on both social and non-social cognitive abilities. This study provide a compelling basis and interesting perspective for future neuroscience work of TCDM behaviors.

## Introduction

Cooperation refers to the behavior/intention of individuals or groups to collaborative in order to achieve a common goal and to promote an outcome that is both beneficial to themselves and to others (Decety et al., 2004). Previous studies have mainly focused on cooperative behavior in social dilemmas, e.g., “joint decision-making” behavior (hereafter JDM), where each individual receives a higher payoff for defecting than for cooperating, but as a whole, are better off if they choose to cooperate (Dawes and Messick, 2000). Nevertheless, in real life, social dilemmas are mostly deliberated and resolved through team/group patterns where most of the decisions are made by more than two people. This type of decision has been defined as team/group collaborative decision-making (TCDM) (Bahrami et al., 2010), Although the goals of TCDM and JDM are to obtain the benefit of cooperation, TCDM does not have contradictions between individual interests and team interests (the goals and the interests of individuals in one team are the same), while in JDM behavior, individuals may give up cooperation within the team in order to pursue a higher payoff.

Previous research has paid extensive attention to JDM behavior. Researchers have unraveled the related cortical regions of cooperative behavior in social dilemmas situations based on abundance researches. In these studies, prefrontal regions, orbitofrontal cortex (OFC), ventromedial prefrontal cortex (VMPFC), dorsal medial prefrontal cortex (DMPFC), right dorsal lateral prefrontal cortex (DLPFC), left parietal operculum and anterior cingulate cortex (ACC), were most commonly reported (Rilling et al., 2002, 2008; Sanfey et al., 2003). However, previous study only focused on the exploration of JDM behavior in single brain frameworks, where solitary participants cooperate with a computer or a person via internet. The information processing across the two interacting brains is thus out of reach from the single-brain frame studies (Hasson et al., 2012; Liu and Pelowski, 2014).

With the development of the hyperscanning technique, the research tendency of cooperative behavior has shifted from experimental single-brain, to a natural multi-brain framework (Hasson et al., 2012; Schilbach et al., 2013). These studies have revealed increased synchronized activity in the right superior frontal cortices and the medial prefrontal region when performing joint actions (Funane et al., 2011; Cui et al., 2012; Dommer et al., 2012), the right temporo-parietal junction (TPJ) during face to face economic cooperation (Tang et al., 2016), and anterior cingulate cortex and prefrontal areas synchrony, between brains of dyad teams playing the prisoner’s dilemma game (PDG) (Astolfi et al., 2011).

Previous studies on TCDM are mostly from the perspective of management, which fully investigated the influence of the attributes and composition of team members on TCDM, such as the influence of emotional intelligence, psychological safety of individual, status difference and the number of team members on TCDM (Carton and Cummings, 2012, 2013; Awino, 2013; Owens et al., 2015; Zhou et al., 2020). Still, a brain-based model that informs how collaborative-team interaction contributes and impacts the outcome of decision- making is needed.

Here, we studied the neural mechanisms of cooperative decision-making behavior, especially TCDM behavior in a two-person interaction, via functional near-infrared spectroscopy (fNIRS) based hyperscanning. Our experimental paradigm adopts an improved dilemma-game paradigm; two participants sitting side by side act as collaborators to play a computer-based PDG, which was composed of individual decision-making (ISDM) stages and TCDM stages. The effects of the different stages, sex and incentive levels were assessed.

Therefore, for the present study, we had three following hypothesis:

H1: The context of decision patterns (individual and team decision stages) may providing some evidence for intra- and inter-brain neural substrate differences between ISDM and TCDM behavior.

H2: Previous researches have confirmed the impact of social environmental cues (e.g., incentive levels) (Vlaev and Chater, 2006), which may also modulated the neural substrate differences between ISDM and TCDM behavior.

H3: Meanwhile, male and female participants may exhibit different neural patterns during ISDM and TCDM processes.

## Materials and Methods

### Participants

We recruited sixty-two healthy, right-handed students from several universities (32 males and 30 females, mean age = 22.3 ± 2.4 years). All subjects were strangers and participated in same-sex pairs (total of thirty-one pairs). Informed written consent was collected from all participants. All experiments were approved by the Southeast University Institutional Review Board.

### Experimental Procedure

**FIGURE 1:**
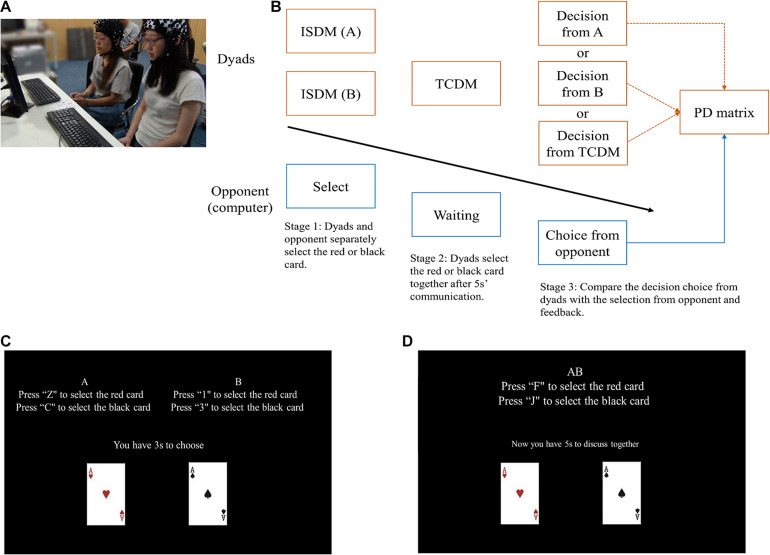
**(A)** Experimental condition. Two participants sitting side by side act as cooperators playing a computer based PDG. **(B)** Main experimental procedure. The orange boxes represent the actions of dyads while the blue boxes represent the actions of the computer. The three orange arrows mean the E-Prime program will choose a choice scheme randomly from choice A, choice B or choice AB to compare with choice C. **(C,D)** Screenshot of the ISDM and TCDM stage.

Two participants (P1 and P2), sitting side by side, act as cooperators playing with a computer. Dyads were told that they need to play with another player in the next room, they do not know their opponent is a computer. We added a cooperative decision-making stage to the classical PDG (Figure 1). The prisoner’s dilemma is a classic game paradigm that studies the conflict between individual interests and groups interests. In the classical two-person PDG, two participants were asked to cooperate or defect with each other, so as to win as many rewards as possible. The reward were classified in to four types, i.e., the reward outcome (R), temptation outcome (T), the sucker outcome (S) and the punishment outcome (P). In each round of the game, if two opponents choose to cooperate with each other, they both receive the reward outcome (R); If one opponent chooses to cooperate and the other chooses to defect, the opponent who defected receives the temptation outcome (T), while the other receives the sucker outcome (S); If two participants choose to defect with each other, they both receive the punishment outcome (P) (Rapoport, 1967). In the present study, Dyad members were asked to choose a red (choice A) or black card (choice B), separately (Participants were informed that the red and black card imply cooperation and defection, respectively). After that, the dyads have to decide together and form a choice scheme for TCDM (choice AB) without language communicate, before all the experiments, we told the dyads to communicate by using hand gestures which were discussed and established during the practical trials. Meanwhile, dyads were told that their opponent (the computer) also made a decision at the same time (choice C). After the two decision-making stages, the E-Prime program will choose a choice scheme randomly from choice A, choice B or choice AB to compare with choice C. It should be noted that the decision of computer is controlled by the E-Prime program, which adopt the tit-for-tat strategy. i.e., always makes the same choice with the participants (the scheme executed in the judging stage in the present study) in the previous trial (Sheldon, 1999).

In order to investigate the effect of social environmental cues, we set up two types of reward outcomes (R). The R for mutual cooperation is 3 yuan (low incentive reward; LIR) or 7 yuan (high incentive reward; HIR), and were performed in a random order. Participants were told that their winnings in each round would be proportionately convert to remuneration at the end of the experiment. e.g., when the uncover result was cooperation (the choice scheme selected and the choice C were both red), P1 and P2 get 3 yuan (China’s currency) together (1.5 yuan each), If the dyad got 280 yuan in total after all the experiments, we will give them 140 (The total reward/2) yuan as the remuneration (70 yuan each, equal to 10.85 USD). The entire experiment consisted of 40 rounds (20 rounds of randomized LIR/HIR tasks), each one lasting 40–50 s. All experimental procedures were implemented using E-prime 2.0 (Psychology Software Tools Inc., Pittsburgh, PA, United States). The reward outcome (R), temptation outcome (T), sucker outcome (S) and the punishment outcome (P) of the improved PDG are reported in Table 1.

**TABLE 1:** The PD matrix in the experiment.

Dyads P3	Cooperate (red)	Defect (black)
Cooperate (red)	3/7 3/7	10 0
Defect (black)	0 10	0 0

Meanwhile, we prepared a text version of experimental guidance and a video of experimental instructions to help participants understand the experimental process. After the practice experiment, in order to establish a baseline for the data analysis, the dyads were asked to rest for 60-s, during which they were required to relax mind and keep motionless as much as possible (Jiang et al., 2015). Formal experiment will start until all participants were familiar with the rules of the game.

By focusing on the two stage of decision actions, the improved PDG in the present study provided an opportunity to assess the behavioral and neural difference of ISDM and TCDM behavior in human-to-human interaction context. Moreover, the design of experiment setting of HIR and LIR, and the team composition of same sex allow us to evaluate the influence of social environmental cues and sex effect.

### Apparatus

**FIGURE 2:**
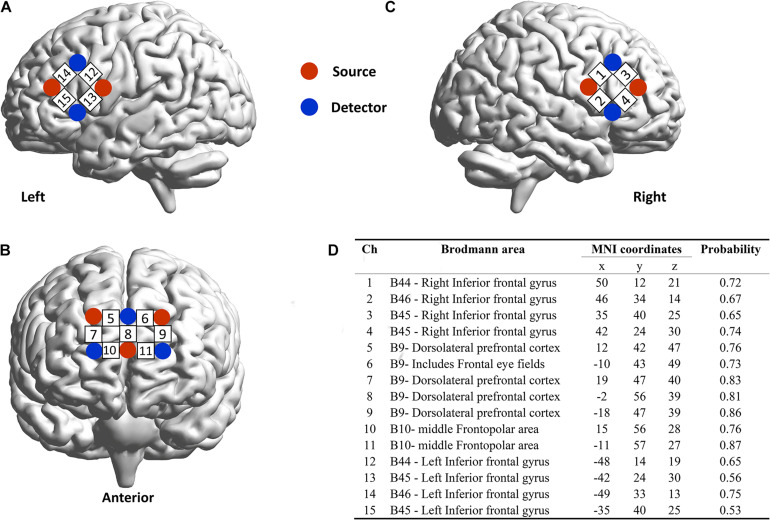
Positions of functional near-infrared spectroscopy (fNIRS) channels. **(A)** front, **(B)** left, and **(C)** right view of fNIRS channels placed on one of the participants in the dyad. **(D)** The Montreal Neurological Institute brain spacing of fNIRS channels.

We used a 16 emitters -16 detectors fNIRS system (LABNIRS; Shimadzu Co., Japan) operated at 780, 805, and 830 nm wavelengths, which could simultaneously measure relative changes in concentrations of oxygenated hemoglobin (Oxy-Hb), deoxygenated hemoglobin (Deoxy-Hb), and total hemoglobin. The relative brain activation was assessed via the conversion of light intensity signals using the modified Beer-Lambert law (Cope et al., 1988). For each participant, one “3 × 3” (forming 12 measurement channels) and two “2 × 2” (forming 8 measurement channels) measurement patches were placed on the prefrontal cortex (PFC) and bilateral IFG, respectively. The center point of each emitter-detector pair (located 3 cm apart) was defined as the measurement channel (we provide a video of the precise positions of the fNIRS channels in Supplementary Materials). The precise positions of the fNIRS channels was measured by a 3D electromagnetic tracking device (FASTRAK; Polhemus, United States) and registered on the Montreal neurological Institute (MNI) brain space using a virtual registration method (Figure 2) (Zhang et al., 2021). The sampling rate is 42 Hz.

### Data Analysis

We used the data preprocessing program in LABNIRS system to remove longitudinal signal drift, motion artifacts, and physiological noise from the raw data, a 0.01–0.1 Hz band-pass filter was applied. Then, a linear baseline correction was performed on the filtered data using the mean value of the Oxy-Hb during the last 10 s of the pre-task period. The fNIRS data were further divided into three stages, ISDM, TCDM, and feedback. Here, we mainly focused on two decision stages. Actions by subject dyads were classified as cooperation or defection, based upon the card chosen. Note that we only focused on the mutual cooperation and defection decisions; in the ISDM stage, the dyads may choose differently, e.g., one chose red while the other chose black. This situation was not considered in this study. Finally, the average group data were calculated from all these categories. The significant level was set at *p* < 0.05. False discovery rate correction was used to minimize false positive results. We primarily focused on the oxy-Hb data, since the oxygenated signal was more sensitive to changes in cerebral blood flow (Hoshi, 2003; Lindenberger et al., 2009). The wavelet coherence between the activations of each dyad was evaluated to examine the inter-brain coupling in each stage (Grinsted et al., 2004).

## Results

### Behavioral Data

In order to examine the effects of sex, task-type and decision-type on the reaction times (RTs) and reaction choices, three-factor repeated measures ANOVA [sex (male vs. female) × decision type (cooperation vs. defection) × task type (low incentive task vs. high incentive task)] were conducted on the behavioral data (i.e., RT and reaction choice).

For the reaction choices, there was a significant interaction between decision-type and task-type during the ISDM stage [*F*(1, 60) = 7.514, *p* = 0.003, η*_*p*_*^2^ = 0.326]. Simple effect analysis revealed that participants formed more cooperative decisions than competitive decisions under the HIR task. There was an interaction between sex and decision-type [*F*(1, 60) = 4.326, *p* = 0.012, η*_*p*_*^2^ = 0.245]. Simple effect analysis revealed that male participants formed more defection decisions than cooperation decisions. There were no significant main effects or other interaction effects (*Ps*>0.05). There was no significant different in RTs.

In the TCDM stage, for the reaction choices, there was a significant main effect of the decision-type [*F*(1, 29) = 9.675, *p* < 0.001, η*_*p*_*^2^ = 0.341]. participants formed more cooperation decision than competition decision. For the RTs, decision-type had a significant main effect [*F*(1, 29) = 7.237, *p* = 0.004, η*_*p*_*^2^ = 0.306]. The average RTs for cooperation decision is shorter than the defection decision. There were no significant main effects of sex, task type or other interaction effects (*Ps* > 0.05).

### Near-Infrared Spectroscopy Data

#### Intra-Brain Activation Differences

We conducted three-factor, repeated measures ANOVA [sex (male vs. female) × decision-type (cooperation vs. competition) × task-type (low incentive task vs. high incentive task)] on the activation values from all dyads.

**FIGURE 3:**
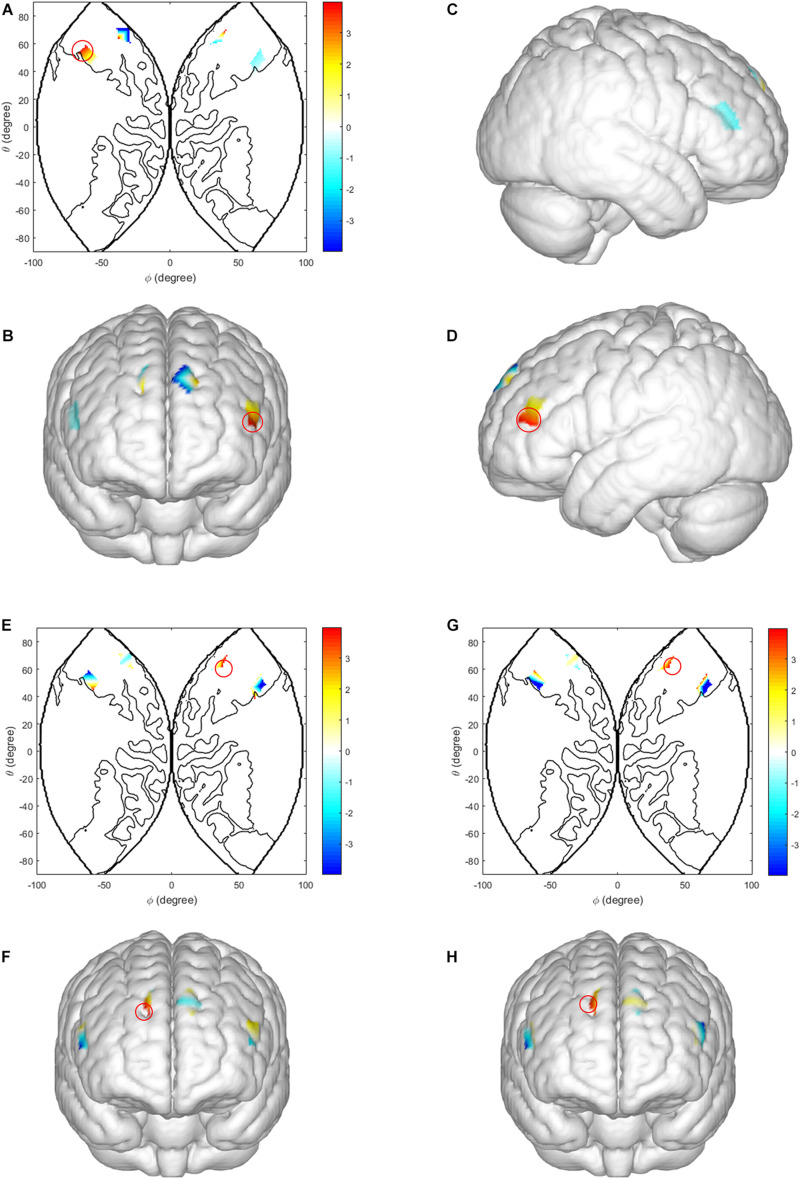
Intra-brain activation in ISDM stage. **(A–D)** Higher activations in the left IFG (LIR condition vs. HIR condition). **(A)** The 2D model of activation map of the left IFG; **(B)** front, **(C)** right, and **(D)** left view of a 3D model of activation map of the left IFG. **(E,F)** Higher activations in the DLPFC and the middle frontopolar area of males (LIR condition vs. HIR condition, when male dyads made defection decision). **(G,H)** Higher activations in the DLPFC and the middle frontopolar area of males (cooperation decision vs. defection decision under HIR condition). Color bar indicated the pared-samples *T*-test value, the red circle represents the statistical significance areas, similarly hereinafter.

In the ISDM stage, we analyzed the intra-brain activation between two subjects in one dyad. We observed a main effect of task-type in the left IFG [CH 14: *F*(1, 60) = 8.241, *p* = 0.010, η*_*p*_*^2^ = 0.314; CH 15: *F*(1, 60) = 7.282, *p* = 0.015, η*_*p*_*^2^ = 0.288, FDR corrected]. The LIR condition evoked higher activations than the HIR condition. There were also significant interaction effects between decision-type, task-type and sex in the DLPFC [CH 7: *F*(1, 60) = 6.132, *p* = 0.01, η*_*p*_*^2^ = 0.419, FDR corrected] and the middle frontopolar area [CH10: *F*(1, 60) = 6.709, *p* = 0.007, η*_*p*_*^2^ = 0.441, FDR corrected]. Simple effect analysis revealed that under the HIR task, there was a significantly higher activation in cooperation decision than defection decision, in male dyads [DLPFC (CH 7): *p* = 0.015; middle frontopolar area (CH 10): *p* = 0.009]; when male dyads made defection decisions, there was a significantly higher activation in LIR condition than HIR condition [DLPFC (CH 7): *p* = 0.017; middle frontopolar area (CH 10): *p* = 0.011] (see Figure 3).

**FIGURE 4:**
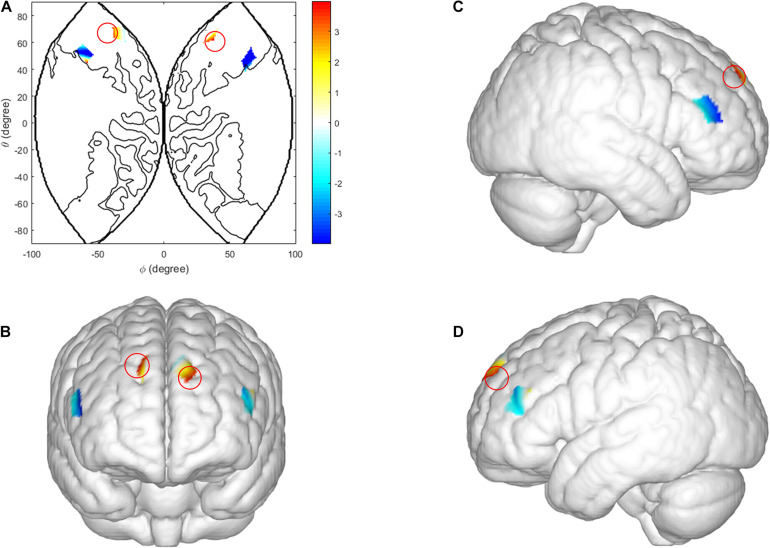
Intra-brain activation in the TCDM stage. **(A–D)** Higher activations in the DLPFC and the middle frontopolar area (LIR condition vs. HIR condition). **(A)** The 2D model of activation map of the DLPFC and the middle frontopolar area; **(B)** front, **(C)** right, and **(D)** left view of a 3D model of activation map of the DLPFC and the middle frontopolar area.

In the TCDM stage, there was a main effect of the task-type in the DLPFC [CH 5: *F*(1, 29) = 7.889, *p* = 0.009, η*_*p*_*^2^ = 0.220; CH 7: *F*(1, 29) = 11.105, *p* = 0.002, η*_*p*_*^2^ = 0.284; CH 9: *F*(1, 29) = 8.088, *p* = 0.008, η*_*p*_*^2^ = 0.224, FDR corrected] and the middle frontopolar area [CH 11: *F*(1, 29) = 9.049, *p* = 0.006, η*_*p*_*^2^ = 0.244, FDR corrected]. Activation of the LIR task was significantly higher than in the HIR task, in the DLPFC and the middle frontopolar area (see Figure 4).

#### Inter-Brain Synchrony

The length of defined interaction behaviors (decision-making) was a minimum of 20 s, and the length of one trial was about 50 s, so we identified a frequency band between 0.02 and 0.05 Hz, where the task occurred, and removed high- and low-frequency noise.

**FIGURE 5:**
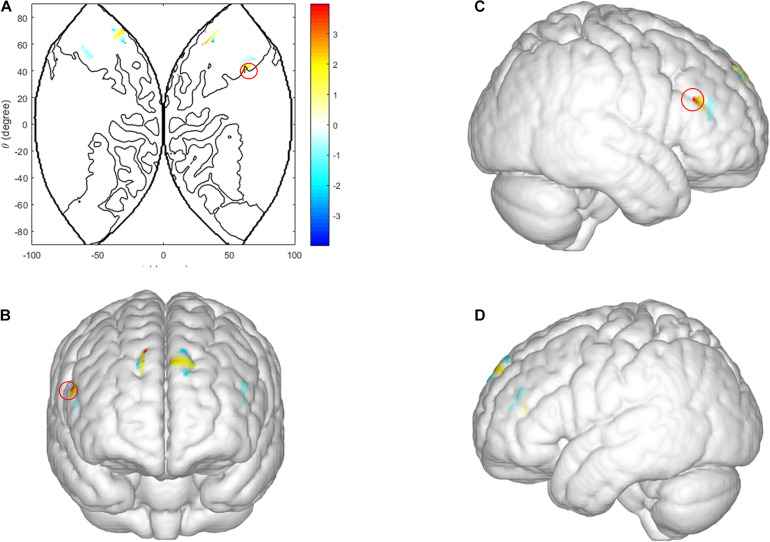
Inter brain synchronization in ISDM stage. **(A–D)** Higher INS values in the right IFG (female dyads vs. male dyads). **(A)** The 2D model of INS values of the right IFG; **(B)** front, **(C)** right, and **(D)** left view of a 3D model of INS values of the right IFG.

Three factor repeated measures ANOVA [sex (male vs. female) × behavior-type (cooperation vs. competition) × task-type (low incentive task vs. high incentive task)] was conducted on the coherence values of all scalp channels, from all dyads. In the ISDM stages there was a significant main effect of sex in the right IFG [CH1: *F*(1, 29) = 7.876, *p* = 0.014, η*_*p*_*^2^ = 0.353]. Female dyads yielded a significant coherence increase compared with male dyads (see Figure 5).

**FIGURE 6:**
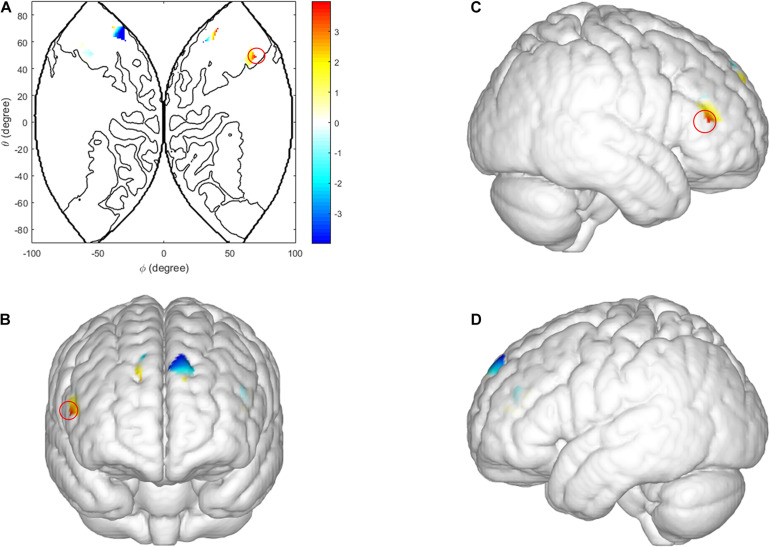
Inter brain synchronization in TCDM stage. **(A–D)** Higher INS values in the right IFG (HIR condition vs. LIR condition). **(A)** The 2D model of INS values of the right IFG; **(B)** front, **(C)** right, and **(D)** left view of a 3D model of INS values of the right IFG.

In the TCDM stages, there was a significant main effect of task-type in the right IFG [CH 2: *F*(1, 29) = 9.985, *p* = 0.004, η*_*p*_*^2^ = 0.263]. The coherence value under the HIR task is significantly higher than the LIR task (see Figure 6).

## Discussion

The experiment performed herein indicate that: (1) Cooperation is a common strategy adopted by the participants, indicating that they tend to cooperate with their opponents (computers in this experiment) in order to obtain the cooperative reward outcome. (2) The experimental setting of HIR promotes a cooperative outcome. Specifically, participants formed more cooperative decisions than competitive decisions under the HIR task. (3) Males were inclined to form more defection decisions than cooperation decisions. This is consistent with the results of previous research on cooperative behavior in social dilemmas, i.e., in team collaborative behavior, males were more willing to defect their opponent, while females preferred cooperation (Balliet et al., 2011).

Regarding the neural substrates activated during cooperative decision-making, the present study found significant results in intra-brain activation. I.e., The LIR condition yielded higher intra-brain activation than the HIR condition (i.e., ISDM stage: in the left IFG, TCDM stage: In the DLPFC and middle frontopolar area). Regarding the activation of cortices between TCDM and ISDM, previous research has shown that the left IFG was activated in inhibitory processes, including the tendency to inhibit learning from undesirable information. For example, TMS to the left IFG has activity has been shown to release such inhibition, increasing the ability to learn from undesirable information (Acheson and Hagoort, 2013). We presume that in the ISDM stage, during the LIR task, the defection decision may obtain a higher reward and as a result, the individual inhibited their tendency for cooperation in order to satisfy the temptation outcome (T), which leads to higher activation in the left IFG. Meanwhile, Sanfey et al. (2003) found that in an economic game, the DLPFC is primarily responsible for suppressing the negative emotions activated by the insula, thus obtaining a rational economic outcome (Sanfey et al., 2003). The middle frontopolar area plays a central role in higher cognitive functions such as planning, problem solving, reasoning, and episodic memory retrieval (Braver and Bongiolatti, 2002). Due to the influence of the partner in the dyad, selfish behavior being inhibited and the tendency to cooperate increases, which caused the activation of the DLPFC and middle frontopolar area during the TCDM stage, as opposed to the ISDM stage. Potentially explaining the activation differences caused by social environmental cues during the ISDM and TCDM stages. This activation diversity reveals the different intra-brain neural substrates between ISDM and TCDM.

With respect to the inter-brain neural substrate, the higher IBS during the TCDM (in the right IFG), was not located in the same cortex where we found in the intra-brain activations (in the DLPFC and middle frontopolar area). Previous studies have confirmed that the regions of intra-brain activation and IBS do not coincide (Baker et al., 2016). On the other hand, this discrepancy might be due to the neural substrates of TCDM, in the social interaction contexts. Meanwhile, the IFG, especially the superior inferior frontal, is a core cortex area of the mirror neurons (MNS), which is activated in imitation, language comprehension and interpretation of intentions (Iacoboni et al., 1999; Tettamanti et al., 2005; Iacoboni, 2008), and plays a key role in reward expectation (Sip et al., 2012). Overall, these behavioral results indicate that the HIR rewards involve common goals and fewer self-other differences, suggesting that it is relatively simple to understand the dyad members actions and intentions. Moreover, this difference in IBS values modulated by social environmental cues, only existed during the TCDM stage, which reveals the differences of inter-brain neural mechanisms between TCDM and ISDM.

The results of intra-brain activation and IBS also found significant sex differences, which was modulated by social environmental cues. Overall, males evoked different intra-brain activations in the DLPFC and the middle frontopolar area while females yielded different IBS in the right IFG, during the ISDM stage. Regarding the sex effect in prisoner dilemmas, accumulated studies have indicated that males maintain a better balance between revenue expectation and trust behavior, which allows them to strategically deal the social dilemma situations (Balliet et al., 2011), which may lead to the higher activation of the cooperation decision during the HIR task. Other studies have found that male tend to be individualized, while females show more altruistic behavior because they are naturally more compassionate (Heilman and Chen, 2005). As mentioned earlier, defection decisions may earn higher rewards and this temptation is stronger in LIR conditions. Therefore, males expect to maximize their reward in the LIR task, which results in the higher activation when males made a defection decision (compared with the HIR task). Meanwhile, Declerck et al. (2013) suggested that the motivation for cooperation was modulated by cognitive control systems (located in the prefrontal cortex, i.e., the DLPFC) and social-brain networks (i.e., the TPJ, IFG, mPFC, and the amygdala) (Declerck et al., 2013). They assumed that pro-selfs are more likely to adopt economic rational strategies and rely on cognitive control systems to make decisions, while pro-social individuals are more likely to adopt social rational strategies and dependent on social-brain networks (Balliet et al., 2011). As such, it is not difficult to explain the sex effect between intra- and inter-brain frames found in this study. That said, influenced by different levels of social environmental cues, males tended to form strategic decisions, while females were inclined to establish cooperation and interpersonal promotion, which leads to the significant results that decision-making behavior in males, showed activation differences in the cognitive control system (DLPFC and middle frontopolar area), while a female’s decision-making behavior showed INS increased in the social brain networks (IFG). This was consistent with the hypothesis of Declerck (Declerck et al., 2013).

Finally, it should also be noted that, despite the increasing popularity of hyperscanning tools in social neuroscience research, we should always be aware of limitations and further questions for future research. First, the sample size was relatively small. In spite of this, the intra- and the inter-brain analysis revealed significant differences between individual and team decision-making behaviors. Second, the dyads in the present study were concentrated among the same sex, further study is thus needed to confirm the sex effect in mixed sex dyads. Third, the ecological validity of the present study still cannot completely generalize to a wider range of team decision-making behaviors, but even so, this study provide a compelling basis and interesting perspective for future neuroscience work of TCDM behaviors.

## Conclusion

That said, the present study draws three conclusions. First, ISDM and TCDM behavior yielded different intra- brain activation and inter- brain synchronization patterns in the neural network of cognitive control system and MNS, this indicated the different neural substrate of ISDM and TCDM behavior. Second, social environmental cues modulated different intra brain activation and inter brain synchronization of ISDM and TCDM behavior. These results confirmed the modulation effect of social environmental cues, also conclude new impact model in the human-human interaction frame. Third, the INS results suggest that in ISDM situation, males and females may primarily depend on non-social and social cognitive ability to make decision separately, while in the social interaction situation of TCDM, females may use both social and non-social cognitive abilities.

## Data Availability Statement

The raw data supporting the conclusions of this article will be made available by the authors, without undue reservation.

## Ethics Statement

The studies involving human participants were reviewed and approved by the Southeast University Institutional Review Board. The patients/participants provided their written informed consent to participate in this study. Written informed consent was obtained from the individual(s) for the publication of any potentially identifiable images or data included in this article.

## Author Contributions

MZ and HJ conceived the project, performed the study, and analyzed the data. MZ designed the study. MZ, HJ, and GW wrote and reviewed the manuscript. All authors contributed to the article and approved the submitted version.

## Conflict of Interest

The authors declare that the research was conducted in the absence of any commercial or financial relationships that could be construed as a potential conflict of interest.
